# Investigation of novel chemotherapeutics for feline oral squamous cell carcinoma

**DOI:** 10.18632/oncotarget.26006

**Published:** 2018-09-04

**Authors:** Hunter John Piegols, Marilia Takada, Maciej Parys, Thomas Dexheimer, Vilma Yuzbasiyan-Gurkan

**Affiliations:** ^1^ Department of Small Animal Clinical Sciences, College of Veterinary Medicine, Michigan State University, East Lansing, MI, USA; ^2^ Comparative Medicine and Integrative Biology Program, College of Veterinary Medicine, Michigan State University, East Lansing, MI, USA; ^3^ Department of Pharmacology and Toxicology, College of Veterinary Medicine, Michigan State University, East Lansing, MI, USA; ^4^ Current Affiliation: The Royal (Dick) School of Veterinary Studies and the Roslin Institute, Roslin, Midlothian, United Kingdom

**Keywords:** feline, oral, squamous, carcinoma, chemotherapeutics

## Abstract

Feline oral squamous cell carcinomas (FOSCC) are highly aggressive neoplasms with short survival times despite multimodal treatment. FOSCC are similar to squamous cell carcinomas of the head and neck (SCCHN) in humans, which also present therapeutic challenges. The current study was undertaken to identify novel chemotherapeutics using FOSCC cell lines. A high throughput drug screen using 1,952 drugs was performed to identify chemotherapeutics for further investigation. Two of the drugs identified in the drug screen, actinomycin D and methotrexate, and two drugs with similar molecular targets to drugs found to be efficacious in the screening, dinaciclib and flavopiridol, were selected for further investigation. Drug inhibition profiles were generated for each drug and cell line using an MTS assay. In addition, the effects of the drugs of interest on cell cycle progression were analyzed via a propidium iodide DNA labeling assay. Changes in caspase-3/7 activity after treatment with each drug were also determined. The findings demonstrated effectiveness of the drugs at nanomolar concentrations with sensitivity varying across cell lines. With all of the drugs except for actinomycin D, evidence for G1 arrest was found. Dinaciclib and flavopiridol were demonstrated to induce apoptosis. The results of the study suggest that the selected drugs are potential candidates for developing novel chemotherapeutic approaches to FOSCC. Through these studies, novel therapeutic strategies for the treatment of FOSCC can be developed to provide better care for affected cats which can also serve as proof of concept studies to inform translational studies in SCCHN in humans.

## INTRODUCTION

Head and neck cancer in humans comprise the sixth most common cancer worldwide, and approximately 90% are classified as squamous cell carcinomas of the head and neck (SCCHN) [1, 2]. Although the one-year survival time is approximately 83% for oral cavity and pharyngeal tumors, the five-year survival time is only 60% [3]. Poor prognostic factors include bone invasion, recurrent disease, and metastasis [4]. Advanced disease is present in approximately half of patients at the time of diagnosis [5]. Human papillomavirus DNA has been detected in 3.9% of oral cavity cancer biopsies and 18.3% of cancers in the oropharynx in humans [6]. Thus, to date, the vast majority of SCCHN tumors are not associated with papillomavirus infection.

Currently, a multi-model therapeutic approach is used for SCCHN including chemotherapy, radiation therapy, and surgical excision [3]. Chemotherapeutics used for head and neck cancer include the following: cisplatin, paclitaxel, carboplatin, 5-fluorouracil, mitomycin-C, cetuximab, and methotrexate [7, 8]. Analysis of the Cetuximab Plus Radiotherapy Versus Cisplatin Plus Radiotherapy in Locally Advanced Head and Neck Cancer Trial suggested that p16-positive oropharyngeal cancer may be more effectively treated with cisplatin than cetuximab in conjunction with radiotherapy; however, more studies are needed due to limitations of this study such as the small sample size [9]. The clinical trial did not specifically address epidermal growth factor receptor (EGFR) expression; however, EGFR expression is inversely related to p16 expression as well as inversely related to induction chemotherapy response and disease-specific survival [10]. Initial studies indicated the potential use of a cetuximab plus docetaxel, cisplatin, and 5-fluorouracil regimen followed by intensity-modulated radiotherapy for laryngeal and hypopharyngeal squamous cell carcinoma [11]. A regimen consisting of methotrexate, leucovorin, 5-fluorouracil, and cisplatin has been demonstrated to improve survival in patients with metastatic or recurrent head and neck cancer [12]. Methotrexate as a single agent has been considered the standard treatment for many patients with advanced, recurrent, or metastatic SCCHN [8]. Methotrexate is one of the most commonly used agents for palliative care in patients with recurrent SCCHN with a response rate of 8–50% and serves as the standard for comparison in phase III studies [13]. Methotrexate may also be beneficial as a single agent for the treatment of verrucous carcinoma, a non-metastatic variant of well-differentiated squamous cell carcinoma [14]. Leucovorin can be combined with methotrexate to reduce severe systemic toxicity with some protocols [15]. A phase II comparison study, however, demonstrated that concurrent chemoradiotherapy using docetaxel, cisplatin, and 5-fluorouracil had an overall greater response rate and greater complete pathological response in comparison to concurrent chemoradiotherapy with cisplatin, 5-fluorouracil, methotrexate, and leucovorin in SCCHN patients with locally advance disease [16].

There are many similarities between feline oral squamous cell carcinoma (FOSCC) in cats and SCCHN in humans which has led to the proposal that FOSCC may serve as a spontaneous model for SCCHN [4]. Oral cancers represent approximately 10% of all diagnosed feline neoplasms with FOSCC accounting for approximately 60% of all oral cancers in cats [17]. The etiological cause of feline oral FOSCC has not been fully elucidated. Epidemiological surveys have identified several potential environmental risk factors including use of flea collars, consumption of a high canned food diet, and consumption of canned tuna; incidence of FOSCC was also increased with household environmental tobacco smoke exposure but failed to achieve statistical significance [18]. Exposure to environmental risk factors may be facilitated in part by the grooming behavior of cats, potentially increasing the oral dose of environmental carcinogens [17]. These studies, however, are both limited in scope and design. Further investigation will be necessary to demonstrate a causal relationship between such factors and the development of FOSCC and to explore other factors. Feline papillomaviruses 1 and 4, the only feline papillomaviruses known to infect oral tissue, are not frequently present in FOSCC lesions suggesting that, similar to SCCHN, papillomavirus infection is not a common cause of FOSCC [19].

FOSCC, clinically, is an aggressive neoplasia that is locally invasive [20]. Patients with FOSCC often present with advanced local disease [21]. In late stages of the disease, FOSCC can invade into local bone tissue depending on the site of tumor origin and metastasize to regional lymph nodes [4]. Though regional and distant metastasis have been reported, patients typically do not present with or develop detectable metastasis due to death resulting from complications associated with the primary tumor [22]. Prognosis for cats diagnosed with FOSCC is poor; the reported median survival time after presentation is less than two months without treatment [21]. When treatment is pursued, median survival time remains poor ranging from two to ten months regardless of treatment modality pursued [22]. After diagnosis, the one year survival rate for FOSCC is less than 10% [20]. Despite multiple therapeutic options, such as radiation therapy, chemotherapeutics, and surgery, significant progress in increasing patient survival has been limited [22]. To date, the use of chemotherapy as a single modality of treatment for FOSCC has had limited demonstrated efficacy and has not been shown to be beneficial as an adjunctive therapy [23]. Toceranib was well tolerated in the majority of cats when treating for FOSCC with low-grade gastrointestinal side effects most commonly reported; however, survival was not assessed due to lack of data, protocol standardization, and wide variety of concurrent treatments [24]. Thus, it remains unclear if the use of toceranib is truly beneficial for the treatment of FOSCC. Overall, similarities in environmental exposures, in addition to similarities in tumor aggressiveness and difficulty in treatment, may allow spontaneous FOSCC in cats to provide insight into non-papillomavirus-induced SCCHN in humans and assist in translational studies for novel therapies [25].

Novel chemotherapeutic options for feline FOSCC is the focus of this study due to the relatively poor response to current treatments, as well as its potential role as a model for SCCHN. FOSCC has been demonstrated to express epidermal growth factor receptor which may serve as a potential target for future therapeutics [26]. Similarly, cyclooxygenase (COX) 1 and 2 have been demonstrated to be expressed in FOSCC [27]. COX inhibitors may be beneficial as adjunctive therapeutics as seen when meloxicam was combined with zolderonic acid and piroxicam was combined with masitinib, a receptor tyrosine kinase inhibitor [28, 29]. FOSCC was responsive to treatment with etanidazole along with radiation therapy, but the mean survival time remained below four months [30]. Ideally, a novel therapeutic would be cytotoxic to neoplastic cells, increase the radiosensitivity of neoplastic cells, and target putative stem cell fractions. No such drug has been demonstrated to fulfill these criteria for FOSCC based on the current literature available.

The current study sought to gain insight into novel therapeutic options for FOSCC focusing on small molecules that may be repurposed. A high throughput drug screen (HTS) was performed to identify compounds with potential use as a chemotherapeutic for FOSCC using three established FOSCC (kindly provided by Dr. Thomas Rosol at The Ohio State University) as well as feline fibroblasts derived from normal skin tissue as a representative for normal tissue [31–33]. Interestingly, the FOSCC cell lines were not susceptible to many of the drugs, but a few drugs warranted further investigation. These drugs included actinomycin D, methotrexate, and multiple kinase inhibitors [36–38]. The present study demonstrates that actinomycin D, methotrexate, dinaciclib and flavopiridol are effective against FOSCC *in vitro* while nontoxic to normal fibroblasts. These drugs are good candidates for future clinical trials in cats with FOSCC and provide insights for potential treatment options for SCCHN.

## RESULTS AND DISCUSSION

### Identification of candidate drugs for treatment of FOSCC

The HTS of the collection of 1,952 compounds from Prestwick, NCI Oncology, and GSK Kinase libraries was performed at 1 mM of each drug. A concentration of 1 μM was selected to screen for strong candidate drugs as concentrations used for hit discovery with drug screening assays range frequently from 1–10 μM [34]. Candidate drugs were selected from the pool applying a cutoff of >25% inhibition in at least one of the FOSCC cell lines, and <60% inhibition in control fibroblasts. Compounds of unknown mechanisms of action were excluded, as well as those sharing >80% similarity in structure to another compound, yielding 60 compounds for further investigation in a dose response assay setting. Drugs associated with altering the microenvironment, such as non-steroidal anti-inflammatory drugs, were excluded as cell culture does not allow evaluation of such effects [35]. Additional drugs with similar targets from the LOPAC library were included for the dose response assay.

From the total of 60 compounds selected for dose response assay, actinomycin D, methotrexate, GW779439X, and GW778894X had an IC_50_ of less than 100 nM and were potent inhibitors against all three FOSCC cell lines and selected for further study. Actinomycin D inhibits RNA transcription by binding double-stranded DNA at specific locations. [36]. Methotrexate is a dihydrofolate reductase inhibitor, though additional effects such as causing epigenetic modifications have been reported. The potential role for methotrexate for FOSCC is further supported by its use for SCCHN [13]. GW779439X and GW778894X are CDK2/CDK4 inhibitors that are under research by GlaxoSmithKline. A recently published kinome profiling data set, however, suggests that GW779439X may inhibit multiple other kinases in addition to CDK2/CDK4 and may be quite promiscuous with multiple off-target effects [37]. Due to the minimal availability of GW779439X and GW778894X, two other CDK inhibitors with similar mechanisms of action were chosen for further study: dinaciclib (Merck, Kenilworth, NJ) and flavopiridol (Sanofi, Bridgewater, NJ). Dinaciclib is a cyclin-dependent kinase (CDK) inhibitor targeting CDK1, CDK2, CDK5, and CDK9 [38]. Dinaciclib may also act as a bromodomain inhibitor [39]. Flavopiridol acts most strongly on CDK1, CDK2, CDK4, CDK6, and CDK7 [40]. Many small molecules have promiscuous effects, as evidenced by kinome profiling data, so it is difficult to exclude that dinaciclib and flavopiridol may have an effect on FOSCC through additional pathways [37]. Of the four drugs selected for further study, only methotrexate is currently reported for the treatment of SCCHN to the authors’ knowledge; thus, the other three drugs may be of translational value [13]. Additionally, the identification of methotrexate in the HTS may suggest that the other drugs identified via the HTS may be useful in SCCHN, though more research would be needed to validate this claim.

### All three FOSCC cell lines were confirmed to be sensitive to actinomycin D, dinaciclib, flavopiridol, and methotrexate

**Figure 1:**
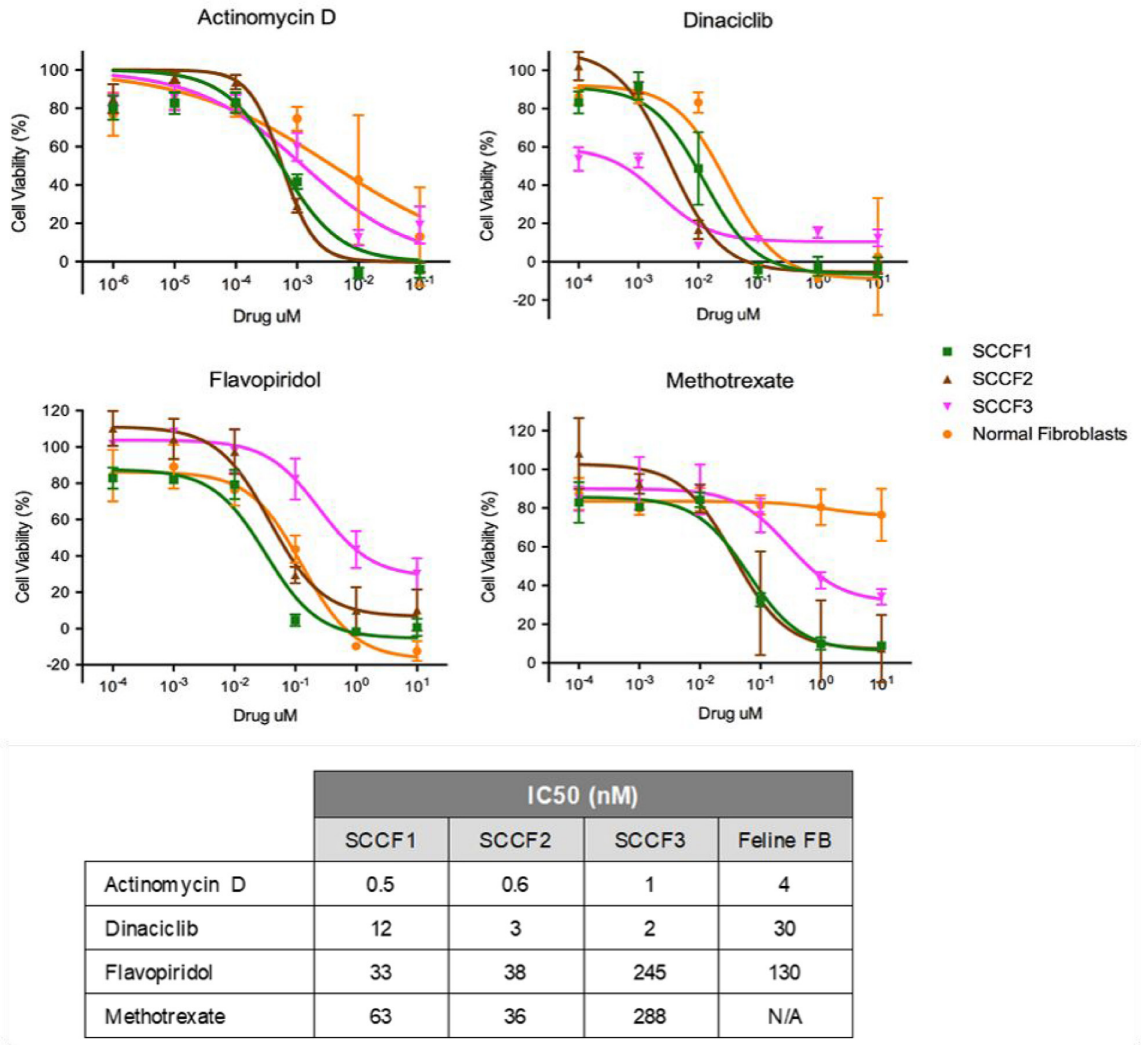
Dose response curves of SCCF1, SCCF2, and SCCF3 cell lines as well as normal feline fibroblasts when treated with actinomycin D, dinaciclib, flavopiridol, and methotrexate Graphs depict MTS assay results plotted as cell viability (%) against each of the drug concentrations. Error bars represent standard deviation. The table contains the corresponding IC_50_ values in nanomolar concentration.

Dose-response curves were performed to confirm the effectiveness of the chosen drugs to inhibit the growth of SCCF1, SCCF2, and SCCF3. IC_50_ values for actinomycin D, dinaciclib, flavopiridol, and methotrexate were extrapolated from the dose-response curves for SCCF1, SCCF2, SCCF3, and primary feline fibroblasts, which were used to reveal potential toxicity to normal cells. The IC_50_ was determined as the concentration in molarity in which the cell viability decreased by 50% as seen on the dose response curves in Figure 1. For additional insight into the achievability of plasma concentrations of the drugs in cats, the IC_50_ values were compared to published values in human pharmacokinetic and toxicity studies. It is important to note that species differences in toxicity and pharmacokinetics exist. For example, cisplatin has been reported to be used at 50–60 mg/m^2^ in dogs for squamous cell carcinoma; however, cats receiving cisplatin at 60 mg/m^2^ are reported to become dyspneic and die within 48–96 hours of administration [41]. Additionally, cats clear some drugs more slowly, such as acetaminophen, propofol, carprofen, and acetylsalicylic acid, than humans and dogs due to relative deficiencies in UDP-glucuronosyltransferase enzymes, N-acetyltransferase, and thiopurine methyltransferase; thus, drugs undergoing metabolism via conjugation may require dosage adjustments in comparison to humans and dogs to avoid toxicity in cats [42]. These differences, however, do not diminish the potential value of using FOSCC as a model for SCCHN as animal models of human disease have been demonstrated to provide translational insight into treatments for human diseases [43].

For all three FOSCC cell lines, actinomycin D had the lowest IC_50_, ranging from 0.6–1.3 nM and an IC_50_ of 4 nM for the feline fibroblasts (Figure 1). With all three cell lines having roughly the same IC_50_ for actinomycin D, sensitivity to actinomycin D appears to be fairly consistent. In a pharmacokinetic study in children being treated with actinomycin D for a variety of tumors, the plasma concentration after 24 hours with variable dosing protocols was 1.8 mM which is higher than the IC_50_ value determined [44]. Dinaciclib had an IC_50_ ranging from 2.5–13 nM for the FOSCC cell lines and an IC_50_ of 32 nM for the feline fibroblasts (Figure 1). Although there is some variation in sensitivity to dinaciclib, the IC_50_ values are relatively similar across all three FOSCC cell lines. Pre-clinical studies in humans suggest that dinaciclib is effective at greater than 50 nM which is achievable at the doses being used in Phase 2 studies; all of the IC_50_ values determined in this study are less than 50 nM [45]. Flavopiridol had an IC_50_ ranging from 32–250 nM for the FOSCC cell lines and an IC_50_ of 130 nM for the feline fibroblasts (Figure 1). There is some variation in IC_50_ values between the three FOSCC cell lines for flavopiridol, though the variation is within one order of magnitude. The IC_50_ value for the least susceptible cell line, however, is less than the steady-state plasma concentration at the maximally tolerated dose reported in Phase I trials [46]. Methotrexate had an IC_50_ ranging from 64–320 nM for the FOSCC cell lines; however, an IC_50_ value was not able to be determined for the feline fibroblasts (Figure 1). There is some variation in IC_50_ values between the three FOSCC cell lines for methotrexate, though the variation is less than an order of magnitude. For the least susceptible cell line, the IC_50_ value is less than 1 μM, the concentration currently considered the maximum concentration 42 hours after the start of a high dose methotrexate infusion to avoid toxicity in people. While the differences are not greater than an order of magnitude, it is interesting that SCCF3 was relatively more sensitive to dinaciclib and less sensitive to flavopiridol and methotrexate than both SCCF1 and SCCF2 (Figure 1). One potential explanation for this is differences in molecular mechanisms driving cellular proliferation as well as differences in metabolic pathways. As a whole, all of the drugs have an IC_50_ value in the low nanomolar range for at least two of the cell lines (Figure 1), supporting the proposed hypothesis. While *in vivo* studies evaluating the pharmacokinetics of these drugs in cats are necessary, the results of this study suggest that effective concentrations may be achievable in the plasma.

By using feline fibroblasts as a surrogate for non-neoplastic tissue, comparisons of IC_50_ values between the cell lines and fibroblasts may provide potential insight into potential concerns about toxicity to the patient. It is important to note that using feline fibroblasts as a surrogate for normal tissue may not be indicative of tissue specific toxicities and further studies into the potential toxic effects of these drugs are warranted; however, the use of feline fibroblasts provides an initial insight into toxic effects. Further studies into potential toxic effects of these drugs are warranted. Actinomycin D had an IC_50_ value for the feline fibroblasts about 7 times greater than the SCCF1 and SCCF2 cell lines and about 3 times greater than SCCF3 cell line (Figure 1). Though the IC_50_ value for actinomycin D for the fibroblasts was relatively low, there was a fairly large difference in IC_50_ values between some of the neoplastic cell lines and the fibroblasts; thus, a very low dose may potentially be given to target neoplastic cells while minimalizing effects on normal tissue. Dinaciclib has an IC_50_ value for fibroblasts greater than for any of the FOSCC cell lines though the difference is greatest for SCCF2 and SCCF3, with the IC_50_ 8 times and 13 times greater for the feline fibroblasts, respectively. There may be concerns about toxicity for treating some tumors; however, there may be subsets of FOSCC that are more sensitive to dinaciclib, especially tumors most similar to SCCF3. For the more susceptible subset, dinaciclib may potentially be a promising treatment option. Further investigation into biomarkers to identify such tumors would be warranted; additionally, the development of a FOSCC drug screen on patient samples may potentially be able to identify useful drugs specific to the patient’s disease. Flavopiridol had an IC_50_ value somewhat greater for the feline fibroblasts than SCCF1 and SCCF2 though the difference was not very large, only about 4 and 3 times greater, respectively (Figure 1). SCCF3, however, had an IC_50_ value nearly double that of the IC_50_ for the fibroblasts. With data from all three cell lines taken into account, flavopiridol may present concerns about toxic effects in normal tissue, and, in some instances, normal tissue may be more susceptible to the drug than neoplastic cells. Nevertheless, flavopiridol may have potential use in some FOSCC patients if developed into an implant for local distribution; similar applications have been used with cisplatin [47]. Methotrexate could not have an IC_50_ value determined for the fibroblasts due to high concentration of drugs that would be required (Figure 1). Similar to actinomycin D, there are fewer concerns about toxicity to normal tissue based on the large difference in IC_50_ values between the fibroblasts and the neoplastic cell lines.

Performing *in vivo* studies pertaining to toxicity as well as pharmacodynamics and pharmacokinetics in cats would add further insight into the potential safety of the selected drugs as *in vitro* analysis does not always represent *in vivo* responses. Until such studies are performed, the comparisons made to pharmacological studies in humans provide insight into whether or not the determined IC_50_ values may be achievable.

### Effects of actinomycin D, dinaciclib, flavopiridol, and methotrexate on cell cycle progression

**Figure 2:**
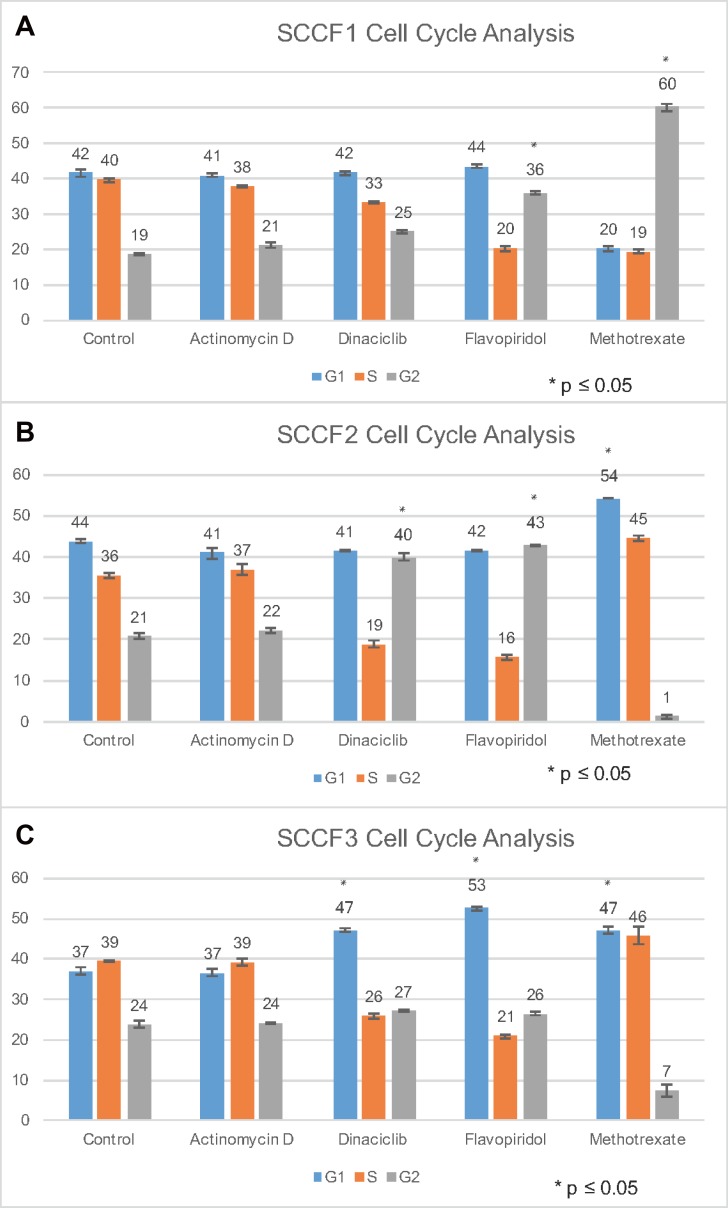
Cell cycle analysis of SCCF1, SCCF2, and SCCF3 cell lines after treatment with actinomycin D, dinaciclib, flavopiridol, and methotrexate Graphs depict percent of cells in G1 phase, S phase, and G2 for the following treatments: control, actinomycin D, dinaciclib, flavopiridol, and methotrexate. (**A**–**C**) represent SCCF1, SCCF2, and SCCF3, respectively. Error bars represent standard deviation.

To gain further insight into the potential mechanism of action of the drugs, a cell cycle assay using propidium iodide was used to determine the effects of actinomycin D, dinaciclib, flavopiridol, and methotrexate on each FOSCC cell line on cell cycle progression (Figure 2). SCCF1, SCCF2, and SCCF3 cells were treated with either actinomycin D, dinaciclib, flavopiridol, or methotrexate for twenty-four hours. Cells were then distributed into G_1_, S, and G_2_ subpopulations with data from a representative experiment displayed (Figure 2 and Supplementary Figure 1).

For SCCF1, actinomycin D and dinaciclib treatment did not greatly alter cell cycle progression (Figure 2A). As the mechanism of action for actinomycin D is through inhibiting RNA synthesis, minimal effect on cell cycle progression is as expected [48, 49]. Flavopiridol had a large shift of cells into G2 for SCCF1, suggesting that these drugs may arrest cells in G2 and prevent progression into M phase, potentially by affecting CDK involved in late G2 or M phase initiation such as CDK1 (Figure 2A) [48]. Methotrexate also had a large shift of cells into G2 for SCCF1, indicative of G2 arrest (Figure 2A). Methotrexate has been previously reported to cause late S/G2 arrest in lymphocytes [50]. Actinomycin D did not greatly alter cell cycle progression for SCCF2 (Figure 2B). Dinaciclib and flavopiridol had a similar effect on cell progression in SCCF2 cells by increasing the proportion of cells in G2 phase (Figure 2B); this may occur through a similar mechanism proposed for SCCF1 for flavopiridol. Methotrexate had a large shift of cells into G1 (Figure 2B). The methotrexate findings are similar to the G1 and S arrest described in human colorectal adenocarcinoma C85 cells when treated with methotrexate; it proposed that the cell cycle arrest was due to DNA damage accumulating, not DNA synthesis inhibition [51]. Similar to SCCF1 and SCCF2, actinomycin D had no significant effect on cell cycle progression for SCCF3 (Figure 2C). Dinaciclib and flavopiridol caused a large shift of cells into G1 for SCCF3 (Figure 2C). Dinaciclib and flavopiridol may arrest cells in G1 and prevent progression into S phase, potentially by affecting CDK involved in late G1 or S phase initiation such as CDK2 for the CDK inhibitors [48]. Methotrexate similarly cause a large increase of cells into G1 for SCCF3; this is most likely similar to the mechanism provided for SCCF2 (Figure 2C). While cell cycle arrest is consistently noted for flavopiridol and methotrexate across all cell lines and for dincaciclib for two of the three cell lines, the effects of the drugs vary depending on the cell line. The mechanism behind this is not well understood. Potential explanations include differences in tumorigenesis and the factors driving cellular proliferation. Further investigation into these changes is warranted in future studies [52, 49]. It is interesting to note that flavopiridol has demonstrated growth suppression of a tumorigenic head and neck squamous cell carcinoma cell line *in vitro* which may further support the finding that flavopiridol alters cell cycle progression [52].

Depending on the stage of the cell cycle in which the drugs inhibit cell cycle progression, radiosensitivity may be altered [53]. Shifts into G1 and G2 phases were noted depending on the drug and cell line (Figure 2A–2C). The shift of cells into G1 or G2 phase may increase the radiosensitivity of the cell lines as cells in G1 are more sensitive to radiation than cells in S phase, and cells are most radiosensitive in G2/M [54]. Thus, inhibition of cell cycle progression has the potential to radiosensitize the FOSCC cell lines, increasing the effectiveness of radiation therapy; however, further investigation is needed to support these claims.

### CDK inhibitors induced apoptosis at higher concentrations across all cell lines

**Figure 3:**
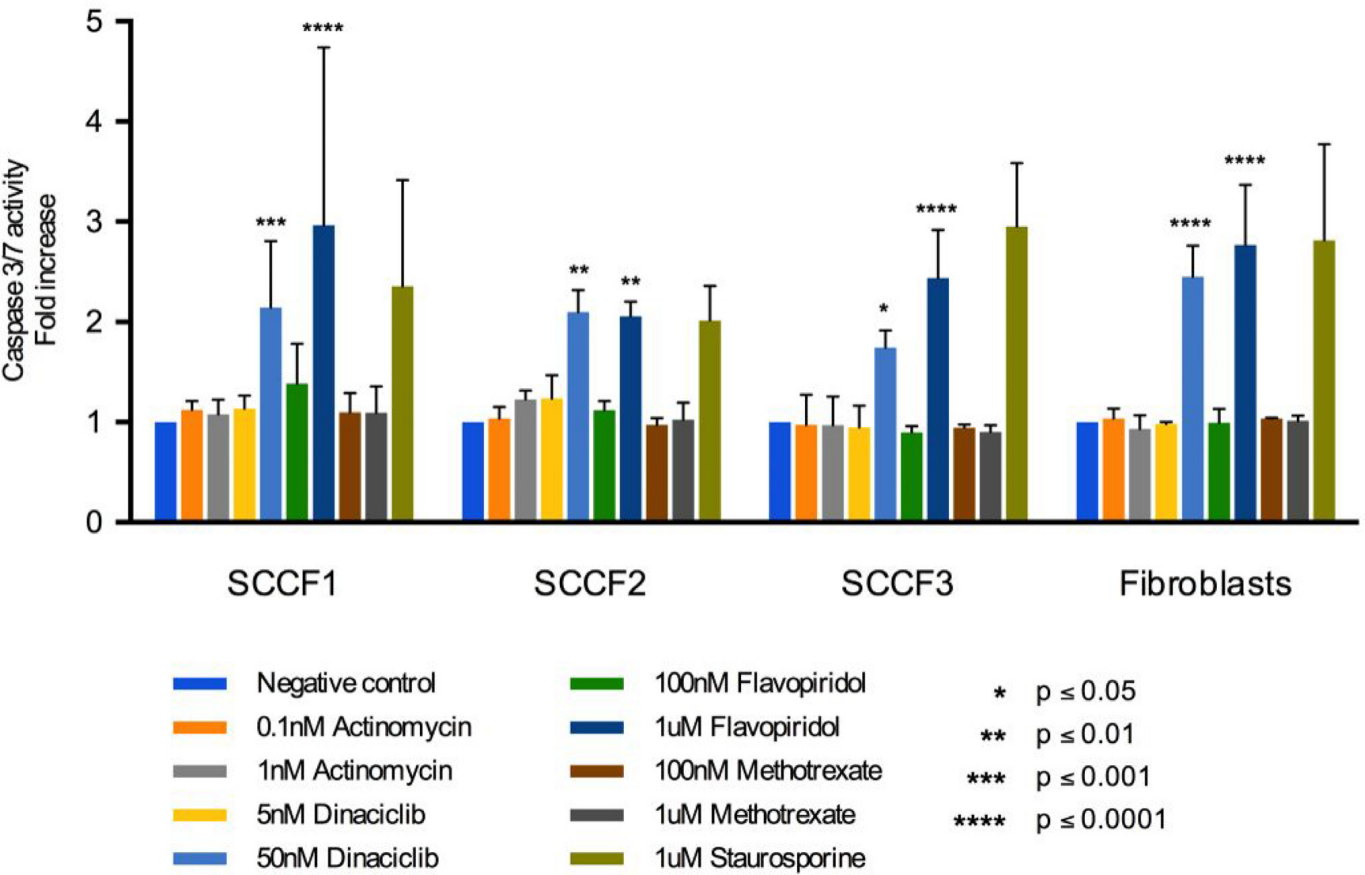
Apoptosis assay for SCCF1, SCCF2, SCCF3, and feline fibroblasts after treatment with actinomycin D, dinaciclib, flavopiridol, and methotrexate for 24 hours Graph depicts fold change in caspase-3/7 activity after treatment with 0.1% DMSO, actinomycin D at 0.1 and 1 nM, dinaciclib at 5 and 50 nM, flavopiridol at 100 nM and 1 µM, methotrexate at 100 nM and 1 µM, and staurosporine at 1 µM. Error bars represent standard deviation.

To determine the ability of the drugs of interest to induce apoptosis in the FOSCC cell lines, the effect of drugs on activity of caspase-3/7 was measured using a luminescence assay. Caspase-3/7 are executioner caspases that cleave many substrates leading to apoptosis [55]. Thus, measuring the activity of caspase-3/7 can act as a surrogate for determining the ability of the drugs of interest to induce apoptosis. SCCF1, SCCF2, SCCF3, and feline fibroblasts were treated with two concentrations of actinomycin D, dinaciclib, flavopiridol, and methotrexate for 24 hours. The concentrations used were approximately the IC_50_ value and ten times the IC_50_ value. Caspase-3/7 activity was greatly increased for dinaciclib and flavopiridol at 50 nM and 1 μM, respectively (Figure 3). Actinomycin D and methotrexate did not induce apoptosis at the low and high concentrations used (Figure 3). Potential explanations include the following: actinomycin D and methotrexate require more than 24 hours to cause cell death or higher concentrations are needed to induce apoptosis within the time frame used. Dinaciclib and flavopiridol did not increase caspase-3/7 activity at 5 nM and 100 nM, respectively, across all cell lines; however, dinaciclib and flavopiridol increased caspase-3/7 activity when used at 50 nM and 1 µM, respectively (Figure 3). Thus, at higher concentrations, dinaciclib and flavopiridol induced apoptosis of all of the FOSCC cell lines. Higher concentrations than the IC_50_ value may be needed because the apoptosis assay was at twenty-four hours while the inhibitory concentrations were determined after treatment for seventy-two hours. The data suggests that the ability of dinaciclib and flavopiridol to induce apoptosis is concentration dependent. One of the goals of chemotherapy for neoplasms is to induce apoptosis due to the correlation between apoptosis and therapeutic response [56]. Thus, the ability of dinaciclib and flavopiridol to induce apoptosis in all three FOSCC cell lines supports the potential of these drugs to be used as chemotherapeutics for FOSCC to target the bulk of the tumor.

## MATERIALS AND METHODS

### Culture of FOSCC cell lines SCCF1, SCCF2, SCCF3, and feline fibroblasts

SCCF1, SCCF2, and SCCF3 cell lines were cultured in DMEM supplemented with 10% fetal bovine serum (FBS), 1% antibiotic-antimycotic (Anti-Anti (100×); Gibco), 0.1% gentamicin (Gibco) as well as epidermal growth factor (EGF) at 10 ng/mL and cholera toxin at 0.1 nM (referred to as complete media). Feline fibroblasts were cultured in DMEM supplemented with 10% FBS, 1% antibiotic-antimycotic, and 0.1% gentamicin (referred to as fibroblast media).

### High throughput screening of FOSCC Cells

Cell lines SCCF1, SCCF2, and SCCF3 as well as feline fibroblasts derived from skin were plated in a 384-well plate at 5,000 cells per well. A total of 1,952 compounds comprised of the Prestwick (Prestwick Chemical), Approved Oncology Drugs Set V (NCI Developmental Therapeutics Program), and Published Kinase Inhibitor Set (PKIS, Structural Genomics Center, University of North Carolina at Chapel Hill) libraries were delivered to cells using a Biomek FX Workstation liquid handling system (Beckman Coulter) using a single concentration of 1 µM. After 48 hours of incubation, CellTiter-Glo Cell Viability (Promega, Madison, WI) reagent was added and luminescence signal was read using a Synergy Neo (BioTek) detection platform.

### Dose response confirmation assay of selected compounds

SCCF1, SCCF2, and SCCF3 cell lines were seeded into 384-well plates at 5,000 cells per well concentration. As cells attached to the bottom of the plate after four hours, selected compounds were added at eight different concentrations with a three-fold difference used and a maximum concentration of 10 µM. Cell viability was measured after 48 hours of incubation as described previously. Using the calculated percentage of viability at each concentration, pIC50 of each compound was calculated using GRETL software within MScreen, an HTS data storage and analysis system (Center for Chemical Genomics, University of Michigan) [59].

### Cell viability assay of confirmed hits

Cell viability assays were performed using SCCF1, SCCF2, and SCCF3 cell lines as well as on feline fibroblasts, which were used as surrogates for normal tissue response to the drugs. The HTS was performed at only one concentration; thus, a dose response curve using a 3-(4,5-dimethylthiazol-2-yl)-5-(3-carboxymethoxyphenyl)-2-(4-sulfophenyl)-2H-tetrazolium (MTS) assay was used to determine the IC_50_ of the drugs of interest. The experiments were performed in triplicate. Cells were plated at a density of 3,000 cells per well in a 96 well plate and then incubated for twenty-four hours. The next day, the media was removed and replaced with complete media or fibroblast media containing either actinomycin D, dinaciclib, flavopiridol, or methotrexate. The following concentrations were used for actinomycin D: 1 pM, 10 pM, 100 pM, 1 nM, 10 nM, and 100 nM. The following concentrations were used for dinaciclib, flavopiridol, and methotrexate: 0.1 nm, 1 nm, 10 nm, 100 nm, 1 μm, and 10 μm. Drugs were dissolved in DMSO with the final concentration of DMSO in all media applied to cells equaling 1%. A vehicle-only treated control consisting of either complete media or fibroblast media with 1% DMSO was used. Plates were then incubated for 72 hours. An MTS assay was then performed using CellTiter 96^®^ AQueous One Solution Cell Proliferation Assay (Promega, Madison, WI), which was incubated for three hours [60]. An MTS assay detects a colored, soluble formazan dye produced by mitrochondrial metabolism of the tetrazolium salt; thus, the detected level of absorbance is proportional to the number of living cells [36]. The results were then determined using EnVision Multilabel Plate Reader (PerkinElmer). The calculated percentage at each (log10) drug concentration was plotted using GraphPad Prism 6 software nonlinear regression curve fitting (GraphPad Software) to calculate the IC_50_ value of each compound.

### Propidium iodide cell cycle analysis

Cell cycle inhibition was analyzed using propidium iodide DNA labelling and flow cytometry as a means to gain insight into a potential mechanism of action [61]. For each of the SCCF1, SCCF2, and SCCF3 cell lines, 5 × 10^5^ cells were added to five wells on a six well plate for each cell line. Plates were then incubated overnight. The media was then removed and replaced with complete media with either 0.1% DMSO, 1 nM actinomycin D, 50 nM dinaciclib, 500 nM flavopiridol, or 5 µM methotrexate. Plates were incubated for 24 hours with the drugs. Cells were then fixed with 70% ethanol and labeled with propidium iodide, and RNAse A was added. Samples were then incubated at 4° C until analysis by flow cytometry. For analysis of DNA content, the flow cytometer BD LSR II (BD Bioscience) was used, and, for data interpretation, MOdFit LT V4.1.7 software (VSH) with auto fit and auto linearity settings was used. The experiment was performed in triplicate. Statistical analysis was done using Tukey’s multiple comparison two-way ANOVA test.

### Apoptosis assay

A luminescent caspase-3/7 assay (Caspase-Glo^®^ 3/7 Assay, Promega) was performed to determine the ability of the drugs to induce apoptosis *in vitro* [62]. For each of the SCCF1, SCCF2, and SCCF3 cell lines as well as the feline fibroblasts, 10,000 cells were plated in a 96 well plate. The cells were then incubated until the cells adhered. The media was then removed and replaced with either 0.1% DMSO, 1 μM staurosporine, actinomycin D at 0.1 nM or 1 nM, dinaciclib at 5 nM or 50 nM, flavopiridol at 100 nM or 1 μM, or methotrexate at 100 nM or 1 μM. Staurosporine, a bacterial alkaloid, was used as a positive control as it is used commonly as an inducer of apoptosis [63]. Cell were then incubated for 24 hours. Caspase reagent from Caspase-Glo^®^ (Promega) was added per well for 30 minutes. Plates were then read using EnVision Plate Reader (PerkinElmer). The experiment was performed in triplicate. Data were statistically analyzed using a multiple comparison two-way ANOVA test.

## CONCLUSIONS

There are multiple possible directions for future studies. Experiments using combinations of the drugs of interest could be performed to evaluate any synergistic effect between the drugs and to explore alternative protocols using lower doses of each drug. Determining the expression of multiple CDK for SCCF1, SCCF2, and SCCF3, as well as with a surrogate for epithelial tissue, would be beneficial by providing insight into the tumorigenesis of these cell lines which would then potentially help further explain the mechanism of action of the drugs. CDK expression could be determined at the transcription level as well as the protein level. In addition, testing the ability of the drugs to target putative stem cell fractions as well as the ability of the drugs to increase the radiosensitivity of the cell lines needs to be performed. Performing studies investigating the safety as well as pharmacokinetics and pharmacodynamics of the drugs of interest in cats are important to optimize a protocol of treatment.

Overall, FOSCC are highly aggressive and invasive tumors in cats [20]. Life expectancy is poor despite treatment, with average life expectancy after clinical presentation ranging from two to ten months despite of treatment [22]. This study demonstrates that actinomycin D, dinaciclib, flavopiridol, and methotrexate are promising therapeutics for FOSCC. All four have IC_50_ values in the low nanomolar range for all cell lines studied: SCCF1, SCCF2, and SCCF3. The IC_50_ concentrations determined may be achievable in the plasma in cats; however, the safety of these drugs as well as investigations into their pharmacokinetics and pharmacodynamics are warranted. The inhibitory concentrations determined are, however, lower than the achievable and tolerated doses for humans, further strengthening the possibility for successful use in cats. [44–46, 57] There does appear to be variability in the sensitivity of the cell lines to the drugs. Intra- and inter-tumor heterogeneity are a normal characteristics of tumors and may explain the differences in susceptibility [58]. The data suggests that the CDK inhibitors may inhibit cell growth by preventing progression through different stages of the cell cycle depending on the cell line. Methotrexate also alters cell cycle progression depending on the cell line. The CDK inhibitors induced apoptosis in all cell lines tested. Thus, initial results suggest that the selected drugs are potential candidates for developing novel chemotherapeutic approaches to feline FOSCC. The use of methotrexate for FOSCC is further supported by the use of methotrexate in SCCHN [13]. In addition to the potential impact on feline health, these studies may ultimately provide translational insight into SCCHN in humans which are equally aggressive cancers in need of improved therapies [4].

## SUPPLEMENTARY MATERIALS FIGURE



## Abbreviations

CDK: cyclin dependent kinase
COX: cyclooxygenase
EGF: epidermal growth factor
EGFR: epidermal growth factor receptor
HTS: high throughput screen
IC_50_: 50% inhibitory concentration
MTS: 3-(4,5-dimethylthiazol-2-yl)-5-(3-carboxymethoxyphenyl)-2-(4-sulfophenyl)-2H-tetrazolium
pIC50: -log(IC50)
FOSCC: feline oral squamous cell carcinoma
SCCHN: squamous cell carcinomas of the head and neck
