# Supplementary material for: Investigation of novel chemotherapeutics for feline oral squamous cell carcinoma
Source: Oncotarget. 2018 Sep 4;9(69):33098–109. doi: 10.18632/oncotarget.26006 (PMC6145701; doi:10.18632/oncotarget.26006)
Supplement: Supplementary file 1 [file oncotarget-09-33098-s001.pdf]

Investigation of novel chemotherapeutics for feline oral squamous cell carcinoma

SUPPLEMENTARY MATERIALS

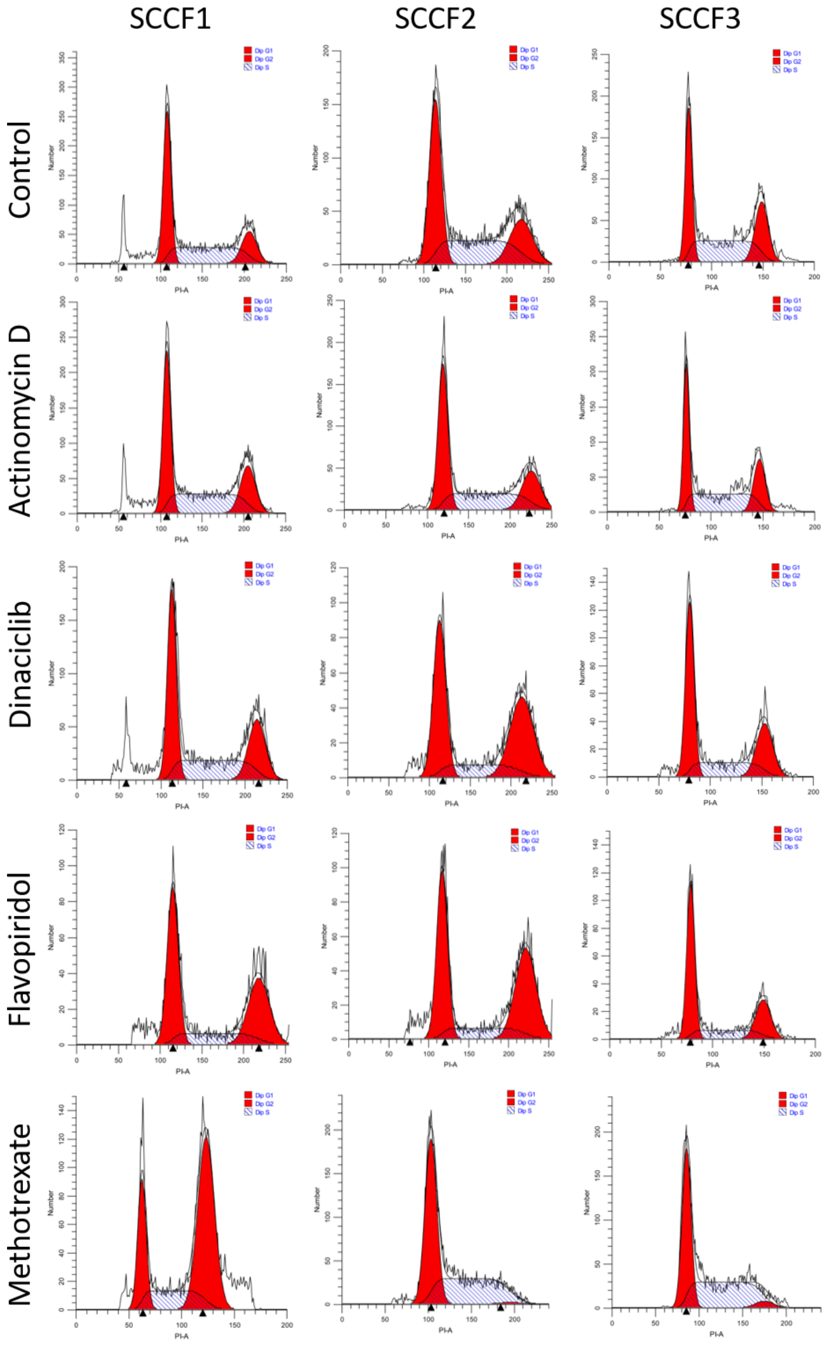

Supplemental Figure 1: Flow cytometry data for SCCF1, SCCF2, and SCCF3 after treatment with actinomycin D, dinaciclib, flavopiridol, and methotrexate. Histogram depicts results of flow cytometry after DNA labelling with propidium iodide. Histogram shows cell count against fluorescence intensity of propidium iodide (PI-A). Three populations were identified: G1 (Dip G1), S (Dip S), and G2 (Dip G2). Data depicts a representative data set.
